# The links between adipose tissue DNA methylation, obesity, and insulin resistance: A protocol for systematic review

**DOI:** 10.1097/MD.0000000000031261

**Published:** 2022-11-25

**Authors:** Suwen Li, Yan Wang, Zinan Li, Cong Long, Qian Zhou, Qiu Chen

**Affiliations:** a College of Clinical Medicine, Chengdu University of Traditional Chinese Medicine, Chengdu, Sichuan, China; b Department of Endocrinology, Hospital of Chengdu University of Traditional Chinese Medicine, Sichuan, China.

**Keywords:** adipose tissue DNA methylation, brown adipose tissue, DNA methylation, insulin resistance, obesity, type 2 diabetes mellitus, white adipose tissue

## Abstract

Obesity is a metabolic condition brought on by the interplay of hereditary and environmental factors, making it one of the most common diseases in the world. Insulin resistance (IR) and obesity have a close connection and can both be advantageous. One of the main methods of epigenetic regulation is DNA methylation modification. Studies have demonstrated over the past few years that DNA methylation is crucial to the emergence of obesity and DNA methylation can lead to IR. Adipose tissue participates in the physiopathological processes of obesity and IR and functions as an endocrine organ controlling the body’s balanced metabolism, thus, adipose tissue-associated gene DNA methylation affects the development of obesity and IR by influencing the function of adipose tissue. Hence, an explanation of current research on DNA methylation, IR, and obesity, following the most recent developments, exploring changes in DNA methylation in different types of adipose tissue in insulin-resistant patients and obese patients may enable the identification of novel targets in clinical obesity prevention and treatment.

**Method and analysis:**

The following electronic bibliographic databases will be searched from inception for peer-reviewed original research published: MEDLINE (through PubMed), Scopus, and EMBASE. Cochrane Library, Cochrane Clinical Trials Registry, the National Institutes for Health Clinical Trials Registry, and the WHO International Clinical Trials Registry Platform from inception to December 31, 2021 will be conducted. Systematic reviews will adhere to the Preferred Reporting Items for Systematic Reviews and Meta-Analyses reporting guidelines. The development of search strategies will make use of medical issue phrases and keywords associated with DNA methylation, Adipose tissue DNA methylation, obesity, and IR. Identified citations will be independently reviewed by two authors to determine eligibility at the title and abstract level, and then at the full text and data extraction phases. Disagreements and conflicts will be resolved through discussion with a third author. Two authors will extract the necessary data from the included studies independently, and The Cochrane Risk of Bias Assessment Tool will be used to assess the bias of randomized controlled studies, and the Newcastle–Ottawa scale for nonrandomized controlled studies. If the interventions and outcomes evaluated are sufficiently homogeneous, results from subgroups of studies will be pooled together in a meta-analysis.

**Strengths and limitations of this study**

1. To our knowledge, this is the first comprehensive systematic review focused on Adipose tissue DNA methylation and the development of obesity and insulin resistance (IR).
2. The results of this study may provide ideas for humans to reveal the underlying mechanisms of obesity and IR as well as treatment and prevention.
3. The results from this systematic review will provide an overview of what is currently known about the relationship between obesity, IR, and adipose tissue DNA methylation.
4. This study will adhere to globally accepted full systematic review methods for evidence screening, assessment of the risk of bias, and data analysis.
5. Given the broad range of the suggested research issues, it is expected that the data would be heterogeneous, which will be taken into account when interpreting the findings.
6. We searched the database for papers published mainly in English and Chinese may mean that other languages may be ignored and important additional findings are missed.

## 1. Introduction

In recent years, obesity has received increasing attention as an important public health problem affecting global human health. Its occurrence is influenced by various factors such as individual, social, and environmental. It is a major risk factor for type 2 diabetes mellitus (T2DM), cardiovascular disease, metabolic syndrome, chronic kidney disease, hyperlipidemia, hypertension, nonalcoholic fatty liver disease, and many cancers.^[1]^ According to the World Health Organization, body mass index ≥ 30.0 is considered obese status, and the global obesity rate has increased dramatically since 1975, with more than 1.9 billion overweight (body mass index ≥ 25) people over 18 years of age in 2016, of which more than 650 million are obese, which is almost twice as many as in 1975.^[2]^ Meanwhile, global obesity rates increased from 0.7% to 5.6% for girls, 0.9% to 7.8% for boys, and 4.7% to 13.1% for adults between 1975 and 2016.^[3]^ By 2030, it is predicted that 57.8% of the older population would be overweight or obese.^[4]^

Other metabolic diseases that are on the rise globally, like obesity, are type 2 diabetes. In 2017, the International Diabetes Federation published data showing that there are 425 million people with diabetes worldwide, and it is expected that by 2045 there will be nearly 700 million people with diabetes, more than 95% of whom will have type 2 diabetes. In humans, IR is typically characterized as the inability of target organs of insulin action to dispose of blood glucose adequately due to increased plasma insulin concentrations, which limits endogenous glucose production (EGP), lipolysis, and the ability to induce glycogen synthesis.^[5]^ IR is a common feature of both obesity and type 2 diabetes, and obesity is a significant factor in the development of IR in patients with T2DM. Obesity and IR are causally linked and have a close relationship. Obesity usually leads to IR, Some studies have shown that altered secretion of cytokines in obesity such as TNF-α, IL-6, and leptin induce IR in obesity,^[6]^ while adiponectin stimulates insulin sensitivity. Fat particles in muscle and liver tissues of obese patients by inhibiting cellular sensitivity to glycemic stimuli. This results in decreased blood glucose uptake and increased free blood glucose, resulting in hyperglycemia, IR, and apoptosis of β-cells in T2DM patients.^[7]^ Also IR can drive obesity, IR reduces the inhibition of lipolysis in the body, increases the accumulation of triacylglycerols in the body, and the high glucose environment increases serum growth factors and elevates blood lipids.^[8]^ IR reduces the sensitivity of adipose tissue to serum insulin and releases more fatty acids into the circulatory system, resulting in the accumulation of fat in the muscle and liver, which in turn leads to the expansion of adipocytes leading to more severe IR, creating a vicious cycle. Therefore, it is necessary to explore effective prevention and treatment pathways to curb the progress of both.

The pathogenesis of obesity and IR is not yet clear. As research progresses, a large body of evidence suggests that obesity and IR are both metabolic disorders that are caused by the interaction of genetic and environmental factors. Epigenetics has become a hot topic of research because it proposes that gene expression is heritable and induced by the environment, linking the environment to the development of many diseases. Epigenetics is defined as heritage changes in the genome without altering the genomic sequence, including DNA methylation, chromatin modification, and noncoding ribonucleic acids.^[9]^ DNA methylation involves the covalent addition of a methyl group to carbon C5 of cytosine nucleotides to create 5-methylcytosine,^[10]^ and mostly associated with cysteine-phosphate-guanine sites located in the promoter regions.^[11]^ The process is catalyzed by the DNA methyltransferase enzymes, and S-adenosyl-methionine is the methyl donor.^[10]^ Finally, the recruitment of transcriptional corepressors and methyl binding proteins, as well as altering the affinity of transcription factors for DNA, may be used to inhibit transcription.^[11]^ Additionally, since DNA methylation can be reversed, therapeutic targets can rely on the change of DNA methylation modification.^[12]^ Previous studies have revealed the links between DNA methylation, obesity, and IR, previous studies have revealed the links between adipose tissue DNA methylation, obesity, and IR, however, the relationship between diabetes, obesity, and adipose tissue DNA methylation have been less systematically evaluated. Adipose tissue is an important organ for IRstorage and hormone secretion in the body and can be divided into white adipose tissue and brown adipose tissue (BAT) according to its morphology, function, and developmental origin. The varied adipose tissue distribution and its malfunction can have a direct impact on the emergence of metabolic disorders including IR and obesity. Numerous clinical and experimental publications have recently reported a connection between IR and alterations in DNA methylation in obese adipose tissue. Genome-wide DNA methylation of subcutaneous adipose tissue in diabetic and non-diabetic populations in different genetic backgrounds identified genes associated with IR such as IRS1, JAZF1, MARCH 1, and TCF7L2.^[13]^ Studies have also revealed that pre-diabetic patients with IR have a significantly higher visceral adipose tissue and that there is a larger association between visceral adipose tissue and IR than there is between subcutaneous adipose tissue.^[14]^ And in obese people, altered DNA methylation in genes linked to STA may result in a reduction in lipid metabolism and inflammation.^[15–18]^ Given that BAT may generate heat, several researchers are investigating ways to treat metabolic conditions including obesity and IR by controlling changes in the DNA methylation of transcription factors related to BAT.^[19–21]^ Adipose tissue is diverse and widely distributed, DNA methylation is tissue cell-specific, and the pathophysiology of obesity and IR is intricate and variable, Therefore, it is necessary for carrying out a systematic review to help advance the exploration of the pathogenesis of obesity and IR and determine whether adipose tissue DNA methylation can be a risk factor, possible biomarker or prognostic marker for these conditions.

## 2. Objectives

This systematic review aims to examine and synthesize the literature investigating the relationship between obesity, IR, and adipose tissue DNA, and determine whether adipose tissue DNA methylation can be a risk factor, possible biomarker, or prognostic marker for obesity and IR. To provide a strategy for advancing the development and screening of drugs for the treatment of obesity and IR by regulating DNA methylation.

## 3. Methodology and analysis

This protocol was reported using the Preferred Reporting Items for Systematic Review and Meta-Analysis Protocols standard.

### 3.1. Eligibility criteria

**Figure 1.**
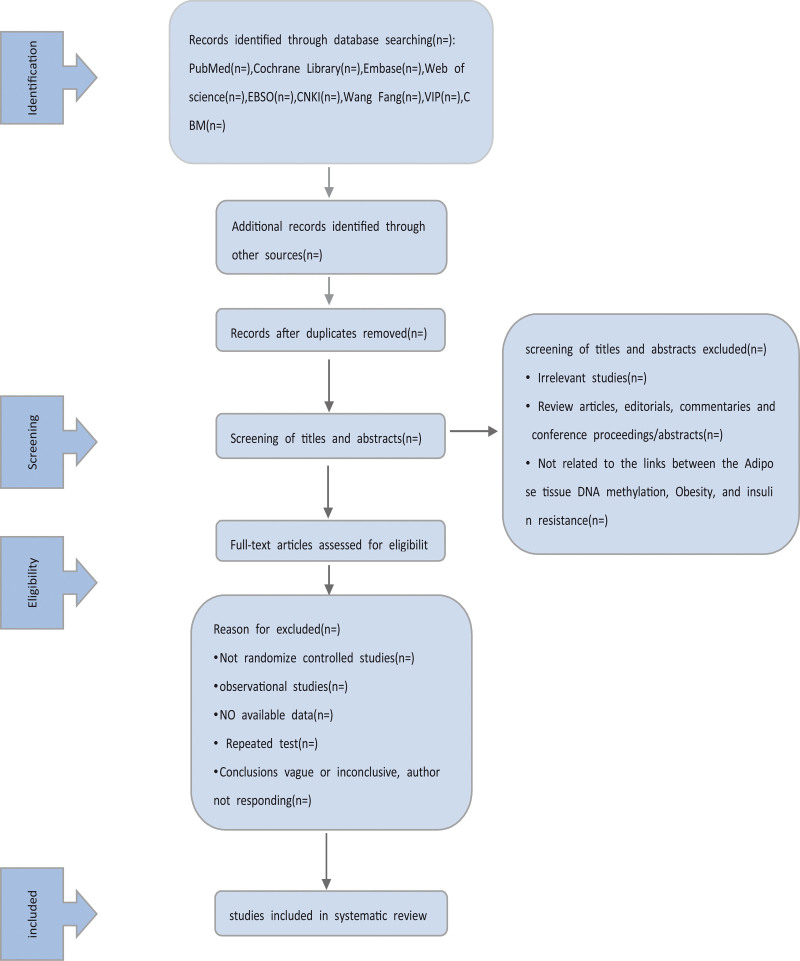
Flow diagram of study selection process.

Randomized control trials and observational studies published in Chinese, and English will be the main subjects of this review. Where applicable, we used meta-analyses and systematic reviews, and we summarized the data that had not been covered by the most recent systematic reviews. Review papers, editorials, commentaries, and conference proceedings/abstracts will not be accepted. The flow chart for systematic reviews clarifies the study screening procedure (Fig. 1).

### 3.2. Information sources and search strategy

The following electronic bibliographic databases will be searched from inception for peer-reviewed original research published: MEDLINE (through PubMed), Scopus, and EMBASE. Cochrane Library, Cochrane Clinical, Trials Registry, the National Institutes for Health Clinical Trials Registry, and the WHO International Clinical Trials Registry Platform from inception to May 31, 2022 will be conducted. The development of search strategies will make use of medical issue phrases and keywords associated with DNA methylation, adipose tissue DNA methylation, obesity, and IR. Table 1 shows the search strategy for one of the included databases. The entire table of search techniques for all databases will be included in the final study. Without regard to language or demographic restrictions, these databases will be searched from their origin to the present.

**Table 1:** Search strategy in PubMed database.

#1	(Obesity) OR (Appetite Depressants) OR (Body Weight) OR (Diet, Reducing) OR (Skinfold Thickness) OR (Lipectomy)) OR (Anti-ObesityAgents) OR (Bariatrics) OR Body mass index)
#2	(insulin resistance) OR (Resistance, Insulin) OR (Insulin Sensitivity) OR (Sensitivity, Insulin)
#3	(DNA methylation) OR (DNA Methylations) OR (Methylation, DNA) OR (Methylations, DNA) OR (Epigenomics) OR (Epigenomic) OR (Epigenetics) OR (Epigenetic).
#4	(DNA methylation Adipose tissue) OR (Adipose tissue DNA methylation) OR (Tissue, Adipose) OR (Fatty Tissue) OR (Tissue, Fatty) OR (Fat Pad) OR (Fat Pads) OR (Pad, Fat)OR (Pads, Fat)OR (Body Fat)
#5	#1AND#2AND#3ADN#4

### 3.3. Data management

At every level of the review, we will manage references using the web tool Cov. The following electronic bibliographic databases will be searched from inception for peer-reviewed original research published: MEDLINE (through PubMed), Scopus, and EMBASE. Cochrane Library, Cochrane Clinical, Trials Registry, the National Institutes for Health Clinical Trials Registry, and the WHO International Clinical Trials Registry Platform from inception to May 31, 2022 will be conducted. The development of search strategies will make use of medical issue phrases and keywords associated with DNA methylation, adipose tissue DNA methylation, obesity, and IR. Table 1 shows the search strategy for one of the included databases. The entire table of search techniques for all databases will be included in the final study. Without regard to language or demographic restrictions, these databases will be searched from their origin to the present. All reviewers will be provided with this methodology and training on how to utilize this tool. We’ll also run a calibration stage using 60 articles to make sure everyone on the team agrees on the review criteria.^[22]^

### 3.4. Selection process

Identified citations will be independently reviewed by two authors to determine eligibility at the title and abstract level, and then at the full text and data extraction phases. Disagreements and conflicts will be resolved through discussion with a third author. There will be a proposed flow diagram for systematic reviews that outlines the justifications for inclusion and exclusion in accordance with the Preferred Reporting Items for Systematic Reviews and Meta-Analyses guidelines.^[23]^ Covidence will be used throughout the study selection process, including finding duplicate studies and organizing the articles.^[22]^

### 3.5. Data collection process

Create and finish the data extraction form on Covidence, which is offered in two versions. This data extraction form includes the following main elements: Authors, nationality, year of publication, journal, study design, study population or species, sample size, sex, biological origin, DNA methylation method, and considering findings from studies on the methylation of DNA worldwide, DNA methylation research’ findings, Additionally, candidate gene methylation studies and GWAS studies will record the types of methylation in tissues and bodily fluids. If the included literature contained ambiguous or inconclusive results or lacking extracted key information, we would contact the authors and retain all correspondence records; if any author did not respond within two weeks, the literature would be excluded.

### 3.6. Risk of bias in individual studies

Systematic reviews will be reported following Preferred Reporting Items for Systematic Reviews and Meta-Analyses guidelines. The Cochrane Risk of Bias Assessment Tool will be used to assess bias in randomized controlled studies.^[24]^ For the observational study, we will use the Newcastle‐Ottawa scale.^[25]^ All assessments will be conducted by two independent reviewers, and any differences and conflicts in assessments will be discussed and resolved with a third reviewer.

### 3.7. Data synthesis

Analysis of statistical data using Review Manager V.5.3. We will use a random effects model to consider the between-study and within-study variance, *I*² statistics, and 95% CI were used to assess heterogeneity, and high heterogeneity will be indicated using the Cochrane recommended *I*² statistic > 40%. The odds ratios and 95% confidence interval were calculated to assess the connections between adipose tissue DNA methylation, obesity, and IR. If the included studies are sufficiently homogeneous (concerning study population, methods, interventions, and outcomes), a random-effects model in STATA version 16 software will be considered to account for meta-analysis of inter-study variability.

### 3.8. Confidence in cumulative evidence

To evaluate the caliber of the evidence, we will grade the suggestions and apply a framework for assessment, development, and review,^[26]^ which evaluate the literature’s consistency, directness, correctness, and risk of bias and publication bias.

## 4. Discussion

A growing number of studies indicates have shown that adipose tissue DNA methylation is closely linked to the development of obesity and IR, however, there is currently insufficient evidence on adipose tissue DNA methylation as an effective therapeutic target for obesity or IR. This systematic review aims to summarize and reassess the data from past studies, investigate the changes in adipose tissue DNA methylation in obesity and IR, and assess whether adipose tissue DNA methylation can be used as a risk factor, possible biomarker, or prognostic marker for obesity and insulin, and to provide new ideas for revealing the underlying mechanisms of obesity and IR and for treatment and prevention.

## Author contributions

All of the authors have agreed to the publication. The review guarantor is Qiu Chen.

**Conceived the idea and Data collection:** Sunwen Li, Yan Wang.

**Designed the study:** Zinan Li, Zhou Qian.**Data curation:** Suwen Li, Qian Zhou, Yan Wang, Zinan Li.

**Formal analysis:** Cong Long, Suwen Li.

**Methodology:** Cong Long, Suwen Li, Zinan Li.

**Software:** Cong Long.

**Supervision:** Qiu Chen.

**Writing – original draft:** Suwen Li, Yan Wang.

**Writing – review & editing:** Sunwen Li, Yan Wang, Zinan Li, Zhou Qian, Cong Long, Qiu Chen.

## Correction

The author order has been changed from “Qiu Chen, MDa, Yan Wang, MMa, Zinan Li, MMa, Cong Long, MDa, Qian Zhou, MMa, Suwen Li, MMb,*” to “Suwen Li, MMa, Yan Wang, MMa, Zinan Li, MMa, Cong Long, MDa, Qian Zhou, MMa, Qiu Chen, MDb,*”. Qiu Chen’s name has been added as the corresponding author.
